# Supplementary material for: Validation study of the Indonesian internet addiction test among adolescents
Source: PLoS One. 2021 Feb 4;16(2):e0245833. doi: 10.1371/journal.pone.0245833 (PMC7861384; doi:10.1371/journal.pone.0245833)
Supplement: S1 File — (PDF) [file pone.0245833.s001.pdf]

INTERNET ADDICTION TEST English Version

This questionnaire consists of 20 statements. After reading each statement carefully, based upon the 5-point Likert scale, please select the response (0, 1, 2, 3, 4 or 5) which best describes you. If two choices seem to apply equally well, circle the choice that best represents how you are most of the time during the past month. Be sure to read all the statements carefully before making your choice. The statements refer to offline situations or actions unless otherwise specified.

0 = Not Applicable1 = Rarely2 = Occasionally3 = Frequently4 = Often5 = Always

1.___How often do you find that you stay online longer than you intended?

2.___How often do you neglect household chores to spend more time online?

3.___How often do you prefer the excitement of the Internet to intimacy with your partner?

4.___How often do you form new relationships with fellow online users? 5.___How often do others in your life complain to you about the amount of time you spend online?

6.___How often do your grades or school work suffer because of the amount of time you spend online?

7.___How often do you check your email before something else that you need to do?

8.___How often does your job performance or productivity suffer because of the Internet?

9.___How often do you become defensive or secretive when anyone asks you what you do online?

10.___How often do you block out disturbing thoughts about your life with soothing thoughts of the Internet?

11.___How often do you find yourself anticipating when you will go online again?

12.___How often do you fear that life without the Internet would be boring, empty, and joyless?

13.___How often do you snap, yell, or act annoyed if someone bothers you while you are online?

14.___How often do you lose sleep due to being online?

15.___How often do you feel preoccupied with the Internet when off-line, or fantasize about being online?

16.___How often do you find yourself saying "just a few more minutes" when online?

17.___How often do you try to cut down the amount of time you spend online and fail?

18.___How often do you try to hide how long you've been online?

19.___How often do you choose to spend more time online over going out with others?

20.___How often do you feel depressed, moody, or nervous when you are off-line, which goes away once

***Internet Addiction Test* (IAT) Indonesian Version**

Internet addiction test (IAT) adalah alat ukur yang terpercaya dan sahih untuk mengukur kecanduan internet. Tes ini terdiri dari 20 butir yang mengukur kecanduan internet dalam derajat ringan, sedang, dan berat. Untuk memulai,

Jawablah pertanyaan-pertanyaan di bawah ini dengan menggunakan skala berikut:

0 = Tidak sesuai; 1 = Jarang; 2 = Kadang-kadang; 3 = Sering; 4 = Sangat sering; 5 = Selalu

1. Seberapa sering Anda mendapatkan diri Anda bermain internet lebih lama dari yang direncanakan?
2. Seberapa sering Anda mengabaikan pekerjaan rumah tangga untuk menghabiskan lebih banyak waktu bermain internet?
3. Seberapa sering Anda memilih kesenangan dengan internet dibandingkan dengan kedekatan bersama keluarga, teman, atau orang terdekat Anda?
4. Seberapa sering Anda menjalin pertemanan baru dengan sesama orang yang bermain internet?
5. Seberapa sering nilai-nilai atau tugas sekolah Anda memburuk akibat jumlah waktu yang Anda habiskan untuk bermain internet?

6. Seberapa sering prestasi sekolah atau tugas Anda memburuk akibat internet?
7. Seberapa sering Anda menutupi atau bersikap rahasia ketika seseorang bertanya apa yang Anda lakukan saat bermain internet?
8. Seberapa sering Anda menutupi pikiran yang mengganggu dengan pikiran yang menyenangkan mengenai internet
9. Seberapa sering Anda mendapatkan diri Anda merencanakan kapan akan bermain internet lagi?
10. Seberapa sering Anda takut bahwa hidup tanpa internet akan membosankan, kosong, dan hilang kegembiraan?
11. Seberapa sering Anda marah, berteriak atau bertingkah mengganggu jika seseorang mengganggu Anda saat bermain internet?
12. Seberapa sering Anda begadang karena online bermain internet hingga larut malam?
13. Seberapa sering Anda merasa terus menerus memikirkan internet ketika sedang offline, atau berkhayal seolah-olah sedang online?
14. Seberapa sering Anda mengatakan "sebentar lagi" saat sedang bermain internet?
15. Seberapa sering Anda berusaha mengurangi waktu yang Anda habiskan untuk bermain internet dan kemudian gagal?
16. Seberapa sering Anda berusaha menyembunyikan berapa lama sebenarnya Anda menggunakan waktu untuk bermain internet?
17. Seberapa sering Anda memilih menggunakan waktu lebih lama untuk bermain internet daripada pergi bersama dengan orang lain?
18. Seberapa sering Anda merasa depresi, labil atau gugup saat offline dan akan hilang ketika kembali bermain internet?
