# Supplementary material for: Validation study of the Indonesian internet addiction test among adolescents
Source: PLoS One. 2021 Feb 4;16(2):e0245833. doi: 10.1371/journal.pone.0245833 (PMC7861384; doi:10.1371/journal.pone.0245833)
Supplement: Attachment — Submitted filename: Responses to Reviewers.docx [file pone.0245833.s002.docx]

Major issues:

| Comments | Responses |
| --- | --- |
| 1- Please explain whether the participants in different stage of process were overlapping. | Thank you for the comment. We have tried to ensure that participants do not overlap in each stage, but there are indeed overlapping participants at the EFA and CFA stages. Although the participants in the CFA did not completely overlap with the EFA, there are new participants included in the CFA. In addition, there is an advantage that these two stages have a large number of participants. |
| 2- Please add the description about the factor structure of the IAT in the instrument section. | Thank you for the input. We have added the description about the factor structure of the IAT in line 107 – 110. |
| 3- The details of the revising process of the IAT need to be provided. For example, how many rounds of the “face validity” did the authors conduct to collect suggestions, revise the test and re-test. | Thank you for the comment. The details of the revising process of the IAT is already written in the manuscript. However, to make it clearer, we have revised it to “one round of item analysis was conducted on 15 JHS students and 15 SHS from seven selected schools through the focus group discussion method to determine the comprehensibility and efficiency of the instructions and terms used in the questionnaire.” (line 125). |
| 4- The factors in a scale are often related. Please use "oblique rotation" and re-conduct the EFA. | Thank you for the comment. We have re-conducted the EFA using oblique rotation (direct oblimin and promax), the result is:  a) direct oblimin  The final result showed IAT consists of only two factors and ten items. The total variance is 45.99% and factor loads ranged from 0.464 – 0.842.  b) promax  The final result showed IAT consists of three factors and fifteen items. The total variance is 49.34% and factor loads ranged from 0.422 – 0.893.  It indicates that EFA using oblique rotation is not any better than EFA we have conducted in the manuscript (using varimax). Therefore, may we be please allowed to keep the EFA result as we have contucted before in our manuscript? |
| 5- It is not recommended to delete the item due to a single reason. Please put all items into the EFA (oblique rotation) and determine whether the items need to be removed. | Thank you for the comment. In accordance with your advice, we have put all items into the EFA using oblique rotation (direct oblimin and promax) and put all items, but we get the same result as before. In both of oblique rotation types, the two items (item 5 and 7) we deleted in the manuscript are still need to be removed (the result showed those items have loading factors <0.4). In addition, the two items we deleted in the manuscript due to the qualitative and quantitative reasons. The quantitative reason is because of the loading factor <0.4, whereas the qualitative reason is already explained in the discussion section (line 285 – 303). Therefore, we decided to delete those items. |
| 6- The explained variance of the EFA is < 60%, indicating that the remained items are not sufficient. | Thank you for the comment. The variance describes how well the items in the IAT measure internet addiction, and the variance will decrease when the item is removed. A variance of less than 60% indicates it is likely that more factors emerged than the expected factors in the model. Therefore, it needs to be accounted for that internet addiction assessment is not only assessed by the IAT. Internet addiction is also influenced by many factors, for example, certain demographic characteristics. In addition, the IAT is not a questionnaire developed in Indonesia so it is possible that the items in the IAT are not sufficient to describe internet addiction in Indonesia when used by Indonesian. We are aware of the limitation of the IAT psychometric in this study, so it is needed to build a questionnaire based on the demographic characteristics in Indonesia. |
| 7- Page 19 Line 295 “for each of the 19 questionnaire items” Please check how many items were used in the process. | Thank you for the comment. We have revised it to “for each of the 18 questionnaire items” (line 236). The Indonesian version of IAT consists of 18 items after going through the reliability and the EFA process. |
| 8- The comparison of factor structure of the IAT in different language versions is essential. Please provide more information about the comparison of factor structure of the IAT in different language versions, especially the item allocation. | Thank you for the comment. We already made the comparison between the Indonesian version of IAT with other different language versions of IAT in the discussion section. However, following your advice, we had added more information about the item allocation (line 315 – 336). We also had compared every item in each domain of Indonesian version of IAT with IAT in different language versions. |

Miner issues:

| Comments | Responses |
| --- | --- |
| 1- There are some typos in the manuscript, such as “the refore”. Please correct them. | Thank you for the input. We have corrected the typos. |
| 2- Page 14 Line175 “The English version of the instrument was subsequently assessed by three experts, including an addiction psychiatrist,…” is confusing, because the authors had translated the IAT to into Indonesian version at the previous stage. Please rephrase the sentence. | Thank you for the input. We have revised it to “The Indonesian version of the instrument was subsequently assessed by three experts, including an addiction psychiatrist,…” (line 118). |
| 3- It is less common to apply a large number of participants in a pilot study. Is there any concern of author to do so? | Thank you for the comment. Yes, it is, because there is an EFA test in the pilot study. The minimal participant used in EFA should meet a rule of thumb for determining sample size, which each item is represented by a minimum of 5 participants. |
| 4- Please change the name “Statistical Package for the Social Sciences (SPSS) version 22”to "IBM Statistical Package for the Social Sciences (SPSS) version 22." | Thank you for the input. We have accordingly changed it (line 142). |
| 5- The “face validity” in this study is more like “item analysis”. Please change the term. | Thank you for the input. We have accordningly changed it (line 34, 96, 125, 154, 176, 177, 199, and in Table 1). |
| 6- Using the internet is not a disease. Therefore, it is inappropriate to say “onset of internet use”. | Thank you for the comment. Appropriate revisions have been made in line 165, 267, 271, 359, 362, and in Table 1. |
