# Supplementary material for: Validation study of the Indonesian internet addiction test among adolescents
Source: PLoS One. 2021 Feb 4;16(2):e0245833. doi: 10.1371/journal.pone.0245833 (PMC7861384; doi:10.1371/journal.pone.0245833)
Supplement: Attachment — Submitted filename: Responses to Reviewers-IAT.docx [file pone.0245833.s003.docx]

| Comments | Responses |
| --- | --- |
| 1- I still think the participants in EFA and CFA should not be the same. If you separate the participants into two groups, will the sample size be too small to conduct the EFA and CFA? If the answer is yes, please add the description about how many participants overlapped in EFA and CFA in the results. Moreover, please add a limitation about this technical issue. | Thank you for the comment. We have added it as a part of our limitations in this study.  Line 368: “Second, there was partial overlapping of participants between EFA and CFA.”  We also have mentioned the number of participants that overlapped in EFA and CFA in the results section.  Line 242-243: “To note, the first 385 respondents in the dataset were overlapping and employed within EFA (N= 385) and CFA (N= 643). |
| 2- Please add a clearly caution that the explained variance of the EFA is < 60%, indicating that the remained items in the IAT are not sufficient. The following users need to interpret the score of IAT very carefully. Adding more items into the IAT is recommended. | Thank you for the input. We have stated it as a limitation within the revised manuscript:  Line 371-373: “Last, the explained variance was less than 60% in the final Indonesian version of IAT, the following users need to interpret the score of IAT very carefully.” |
